# Flexural Behavior of Composite Concrete Slabs Made with Steel and Polypropylene Fibers Reinforced Concrete in the Compression Zone

**DOI:** 10.3390/ma13163616

**Published:** 2020-08-15

**Authors:** Barbara Sadowska-Buraczewska, Małgorzata Szafraniec, Danuta Barnat-Hunek, Grzegorz Łagód

**Affiliations:** 1 Faculty of Civil Engineering and Environmental Sciences, Bialystok University of Technology, Wiejska 45 A, 15-351 Bialystok, Poland; barbara.sadowska@pb.edu.pl; 2 Faculty of Civil Engineering and Architecture, Lublin University of Technology, Nadbystrzycka 40, 20-618 Lublin, Poland; d.barnat-hunek@pollub.pl; 3 Faculty of Environmental Engineering, Lublin University of Technology, Nadbystrzycka 40 B, 20-618 Lublin, Poland; g.lagod@pollub.pl

**Keywords:** deflection, bearing capacity, bending, steel fibers, polypropylene fibers, reinforced concrete slabs, cracks, pores, frost resistance

## Abstract

The paper presented aimed at examining the effect of a fiber-reinforced concrete layer in the compressed zone on the mechanical properties of composite fiber-reinforced concrete slabs. Steel fibers (SF) and polypropylene fibers (PP) in the amount of 1% in relation to the weight of the concrete mix were used as reinforcement fibers. The mixture compositions were developed for the reference concrete, steel fiber concrete and polypropylene fiber concrete. The mechanical properties of the concrete obtained from the designed mixes such as compressive strength, bending strength, modulus of elasticity and frost resistance were tested. The main research elements, i.e., slabs with a reinforced compression zone in the form of a 30 mm layer of concrete with PP or SF were made and tested. The results obtained were compared with a plate made without a strengthening layer. The bending resistance, load capacity and deflection tests were performed on the slabs. A scheme of crack development during the test and a numerical model for the slab element were also devised. The study showed that the composite slabs with fiber-reinforced concrete with PP in the upper layer achieved 12% higher load capacity, with respect to the reference slabs.

## 1. Introduction

The requirements for the load-bearing capacity and serviceability of structures are becoming more and more stringent under various conditions of operation, and meeting them is becoming problematic in the case of ordinary concrete. The reason for the formation and propagation of cracks in concrete structures is their brittleness, which causes the use of normal concrete as a material transmitting tensile stresses independently to be limited [1]. Shrinkage cracking is a major concern when it comes to concrete, especially for flat structures such as garage slabs. This necessitates the search for new types of concrete, the mechanical properties of which will meet the current requirements. One of the methods to reduce the negative effects of shrinkage cracks in addition to classic bar reinforcement is the reinforcement of concrete with short, randomly distributed steel fibers (SF) [2,3,4,5,6,7,8], polypropylene (PP) [2,9,10,11,12] and hybrid fibers [13,14,15]. There are several articles on the quite controversial use of human hair as dispersed concrete reinforcement [16,17].

According to the literature review, the use of different fibers in the concrete technology has become very popular in recent decades. This type of concrete is called fiber reinforced concrete (FRC) and can be defined as a material consisting of Portland cement, fine and coarse aggregates as well as short, irregular fibers. By using FRC, architects and designers have greater architectural freedom in designing the shapes and forms of structural elements because the use of steel reinforcement bars can be almost eliminated. Many factors influence the mechanical properties of the fiber-reinforced concrete [1,2,3,4,5,6,9,12,18,19,20,21,22,23,24,25,26,27,28,29]. These factors include the type of fibers, their geometry, surface structure, quantity in the concrete mix, fibers aspect ratio, mixture design and mixture densification, as well as the laying and curing methods [29,30].

Usually, PP [2,9,12] and SF [2,3,4,5,12,22] are used to improve the mechanical and physical properties, especially tensile and flexural strength as well as long-term concrete shrinkage. The low modulus of elasticity of the PP fibers helps to reduce early shrinkage and control surface concrete chipping during fire [12]. The PP fibers improve the interface condition of aggregate and cement, reduce the formation and development of cracks, as well as decrease the high brittleness of concrete [12,31,32,33]. Unfortunately, the PP fibers also have flaws, decrease the workability of the concrete mixture, reduce the concrete carbonation depth, water permeability, dry shrinkage [12,32,34] and also decrease the frost resistance of concrete by creating voids and pores in the interfacial transition zone (ITZ), which fill with ice during freezing [12,35]. A large amount of PP in the concrete mix causes the formation of larger fiber clusters, which are not evenly distributed in the entire volume of concrete [18,36], contributing to the formation of cracks and gaps between PP and ITZ [12]. Generally, the fibers with a length of more than 40 mm disturb the workability of the concrete mix. The use of liquefying admixtures is recommended in order to improve the workability of the mixture, reduce porosity and minimize the formation of fiber balls. In addition, the PP fibers constitute hydrocarbon polymer materials that are added to cement paste, causing the water layer to settle at the interface between the fibers and the matrix. In view of the above, the authors [12] showed in their microstructural studies that the binding force of PP and matrix surfaces is quite weak. Numerous pores between PP and cement paste lead to a significant increase in water absorption, which was confirmed by Smarzewski and Barnat-Hunek [12]. They showed that the water absorption by ultra-high performance concrete with 1% steel fibers was 100% lower than in the concrete with 1% PP fibers. The portlandite crystals can simply grow, making ITZ more porous. The authors noticed in the SEM images that the PP fibers are mostly broken, and in some cases, they were pulled out from the cement paste without any material damage [12]. This phenomenon significantly reduces the sustainability of FRC. The surface of the SF fibers is rougher than that of the PP fibers. Apart from the reduction of pores in ITZ, this fiber increases the bonding and strength in the ITZ between SF and cement paste, which was confirmed by other investigations [12,22]. The basic material for the production of SF used in fiber concrete is steel, characterized by high yield strength, usually in the range 500–1500 MPa [37]. Fibers can be manufactured using various technologies; however, straight or bent fragments of cold drawn wire have become most popular. The high compressive strength of concrete may be caused by the inclusion of steel fibers in the control of tangential stresses in the triaxial state of stress occurring in compressed concrete cubes. The effect of steel fiber on the compressive strength of concrete is not that significant. However, they greatly contribute in the post-peak region of the load–displacement graph due to the bridging effect. As such, the specimens can undergo larger deformations and the failure changes from brittle to ductile. The authors [12] demonstrated that the splitting tensile strength of the fiber-reinforced concrete increased depending on the content of SF by volume and was higher by 52%, by 0.75% of the SF by volume. Kalpana and Tayu [38] assumed that the expansion of the SF volume portions from 0.5% to 0.75% increased the shear strength by 25–45%. The authors [32] reported that the application of SF to the concrete mixture in the amount of 1–1.5% by volume will increase its bending strength by as much as 150–200%, tensile strength by up to 100%, and compressive strength by 10–25%.

Designers quite often use one of the most innovative structural solutions, namely composite structures. These are structures combined of two materials, e.g., steel–concrete, wood–concrete and concrete–concrete. The combination of two materials is used in the construction of such structural systems as plates, columns, beams and spandrel beam [39,40]. The concrete-to-concrete combination is usually used in prefabrication. These can be reinforced concrete or prefabricated prestressed elements combined with complementary monolithic concrete. Often, the combination of two concretes is used in bridge construction, where the aggressive environment and dynamic loads accelerate the corrosion process of bridge structures. The cooperation of the two concrete layers in a practical way is governed by their interconnection, so-called adhesion [40]. The determination of the joint work of these two materials is directly determined by its deformation. The contact deformation, resulting from the applied loads, is the mutual displacement of the connected elements [41]. According to the literature related to the research carried out on the joint plane of two layers of new and old concretes, it can be concluded that the increase in joint load capacity is proportional to the increase in the strength of the new concrete. The most intensive period of the load capacity increase occurs in the first 7 days of concrete curing. The concrete contact load capacity is determined by the cement hydration process. This means that the specific adhesion has a greater influence here [42]. In this case, the contact load capacity depends on the age of the “young” concrete. The younger it is, the greater the load-bearing capacity of the joint between the “young” concrete and the “new” concrete [40,41].

In this research, the influence of fibers on the mechanical properties of steel or polypropylene FRC composite panels was considered. The lower layer of the slab (5 cm thick) was ordinary concrete, while the upper layer (3 cm thick) was fiber concrete—one with SF and one with PP fibers. Two concretes of different classes and structures were wet-bonded. The purpose of using steel and polypropylene fibers is to control cracks at different curing periods of concrete slabs and different crack widths. In addition, the polypropylene fiber is a rather cheap polymer and can be an effective approach to increase the possibility of plastic shrinkage cracking, impact resistance and hardness of a reinforced concrete fiber slab.

The experimental study consisted of tests on cubes, beams, cylinders and prismatic samples made of ordinary concrete and SF or PP reinforced concrete. Compressive and flexural strength, static modulus of elasticity, frost resistance and tensile flexural behavior, as well as bending resistance test of a complex concrete slab were determined and analyzed. A comparative analysis of the experimental results and results from numerical simulations in terms of deflections was also performed.

## 2. Materials and Methods 

### 2.1. Materials

**Figure 1:**
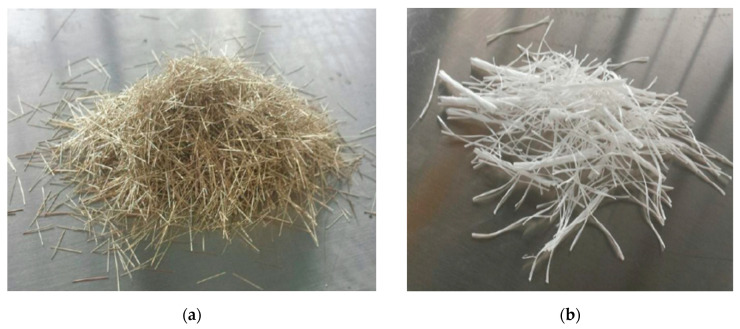
Type of fibers used in conducted studies: (**a**) steel fibers and (**b**) polypropylene fibers.

Two types of fibers were used in the research carried out: straight steel fibers (NV BEKAERT SA Zwevegem, Belgium) and polypropylene fibers (CHRYSO Polska Sp. z o. o., Warsaw, Poland). The appearance of the fibers applied is shown in Figure 1, while their physical and mechanical properties are summarized in Table 1. 

Three concrete compositions for testing were established on the basis of EN 206–1 [43] standard and their contents are shown in Table 2. The concrete samples were marked as follows: C-REF—reference concrete; F-RCS—fiber-reinforced concrete with steel fibers and F-RCP—fiber-reinforced concrete with polypropylene fibers. The fiber-reinforced concretes differed in the type of fiber used. Steel and polypropylene fibers were used in the amount of 1% in relation to the weight of the concrete mix. Apart from that, the concrete compositions were the same when it comes to the type and quantity of components used. As far as the reference concrete is concerned, a different composition of components was used than in the fiber-reinforced concrete. Besides fibers, the mixtures differed in type and content of cement, sand, coarse aggregate and the type of plasticizer used for their production (Table 2). In addition, reactive powder and silica ash were used to make the F-RCS and F-RCP mixtures.

Table 3 and Table 4 show the technical parameters of the cement used in the production of concrete. Except for the individual properties, the tables give the requirements for cement that must be met, based on EN 197-1 [44].

The fine and coarse aggregates used in the concrete mixtures met the provisions of the EN 12620 standard [47].

A very important stage in the production of samples and test elements was the proper design of the concrete mix as well as its preparation. The mixture using steel and polypropylene fiber required proper dosing of ingredients in the right order and mixing time. Weighted components were added in the following order: concrete with dispersed fiber initially dry components were mixed for about 7 min (sand, cement, silica dust and reactive powder), then the fiber was added and mixing continued for another 10 min. After that time, a superplasticizer with 1/3 of the water volume was added to the previously mixed ingredients. After 3–4 min of further mixing the remaining water was added. Then, in order to prepare the samples and elements for testing, the concrete mixture was laid to the previously prepared forms.

As mentioned above, different plasticizers were added to particular concrete mixes. Their characteristics are shown in Table 5 and Table 6 below. Polycarboxylate superplasticizer, which accelerates the concrete hardening, was applied in the F-RCS and F-RCP concrete. This admixture does not contain chlorides or other chemical compounds that cause the corrosion of reinforcing steel. Its application allows for a significant reduction in the amount of water used. This, in turn, enables obtaining high density and strength concrete as well as improves workability. For the C-REF concrete, a standard plasticizing admixture was used. Its application results in the homogenization of the concrete mix and improvement of workability. The use of this plasticizer allows reducing the amount of water added to the concrete mix. The dosage of superplasticizer and plasticizer was 0.8% and 0.7% of cement weight respectively.

According to EN 1008 [48], the water from an available water supply system was used to produce all concrete mixes.

Moreover, reactive powder and silica ash were added to the F-RCS and F-RCP concretes. Their properties are presented in Table 7 and Table 8. The reactive powder was added in the amount of 3.5% of cement mass while the amount of silica dust was 216 kg per m^3^ of the batch of concrete. The use of reactive powder may increase the water content without reducing the strength characteristics of the concrete. This material is a highly reactive pozzolanic additive, which contains the active forms of aluminum and silicon oxides. Its use improves workability, plasticity and consistency of the mix. It is a factor responsible for lowering the temperature dynamics of concrete and increasing the resistance to negative environmental effects. The application of the powder eliminates the sulfate corrosion. In turn, silica dust is a powdered mineral additive with highly pozzolanic properties. Its use reduces lime hydroxide, increases watertightness and improves the workability of the concrete mix.

### 2.2. Methods

Four types of samples were prepared for the tests. For the determination of the compressive strength, 18 cubic samples (6 per concrete) with an edge length of 100 mm were made. Cylindrical specimens with a height of 300 mm and a diameter of 150 mm, in the amount of 3 samples for each concrete, were made to test the static modulus of elasticity (total of 9 samples). The flexural strength test was carried out on 400 mm high prism-shaped samples with the base dimensions of 100 mm × 100 mm (total of 9 samples, 3 for each concrete). The dimensions of the above-mentioned samples were in accordance with the EN 12390-1 standard [51]. Their method of production followed the provisions of the EN 12390-2 standard [52], which specifies the methods of making and curing the samples for concrete strength tests.

The first stage of sample making was to measure the weight of individual components with the accuracy of 0.1 g, according to the recipes given in Table 2. A counter-rotating mixer was used to produce a concrete mix of reference concrete and fiber concrete. Then, the concrete mix was laid into the previously prepared molds and compacted in three stages. The inner surface of the molds was covered with an antiadhesive agent. The vibration was applied for the shortest possible time until the concrete mix was properly compacted. After the mixture was compacted, the excess concrete above the top edge of the mold was removed using a trowel. The surface of the individual samples was then leveled. The samples were subsequently labeled accordingly and left for 24 h at 20 ± 5 °C. After that time, the samples were removed from the molds and placed on a laboratory tray. The samples were cured until laboratory testing in water at 20 ± 2 °C.

**Figure 2:**
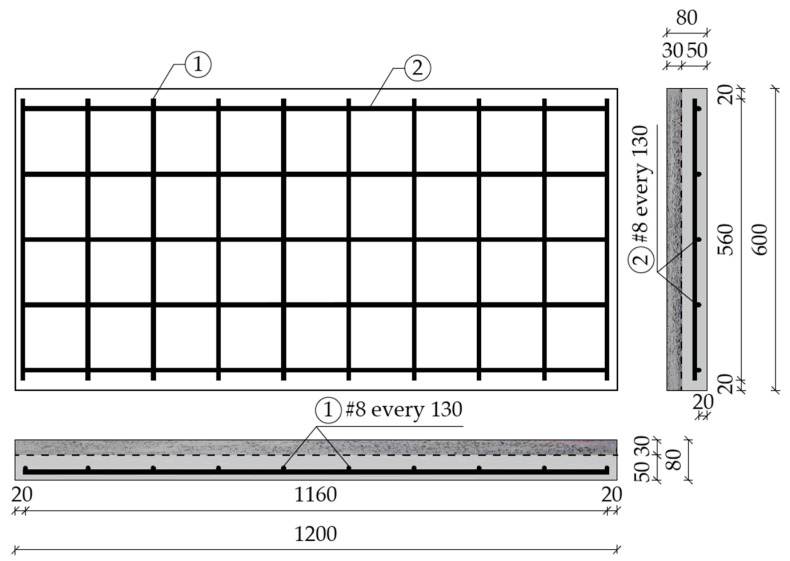
Reinforcement of the slab element.

The main research elements were reinforced concrete slabs made from reference concrete and reference concrete combined with fiber-reinforced concrete. They were used to demonstrate the influence of the application of a layer of fiber concrete in the compression zone on deflections, the nature of crack development and the load-bearing capacity of the slabs. The slabs were marked as follows: C-REF—reference concrete; C-REF + F-RCS—reference concrete combined with the fiber-reinforced concrete with steel fibers and C-REF + F-RCP—reference concrete combined with the fiber-reinforced concrete with polypropylene fibers. For the above-mentioned types of slabs, 3 slabs for each were made (total of 9 slabs). The slabs had dimensions of 1200 mm × 600 mm × 80 mm. In the C-REF + F-RCS and C-REF + F-RCP slabs, the lower layer of 5 cm thick was the reference concrete, the upper layer (3 cm thick) was fiber concrete, while the C-REF slabs were made entirely of the reference concrete. In the C-REF + F-RCS and C-REF + F-RCP slabs, two different types of concrete were “wet combined”. The molds for slabs were made of plywood. The reinforcement of the slabs was a reinforcement net, formed from 8 mm (in diameter) ribbed steel bars of the BS500SP steel grade. The reinforcement was designed in accordance with EN 1992-1-1 [53]. The longitudinal and transverse spacing of the bars was 13 cm. A concrete cover of 20 mm was provided everywhere. The reinforcement and dimension of the slabs are shown in Figure 2.

The molds for the slabs were precoated with an antiadhesive agent. After the concrete mix for the molds had been laid, the lower layer of the test element made of reference concrete was compacted on a vibrating table. The upper layer of the slab, fiber concrete, was also laid and compacted on a vibrating table. Next, the surface of the slabs was leveled. After concreting the slabs, they were left for 24 h at 20 °C. Following their removal from the molds, the main research elements were covered with PVC foil and cured until the strength tests were carried out.

The compressive strength test was carried out on the cubic samples on the basis of standard EN 12390-3 [54] on a testing machine. The cubic sample at the time of loading was turned by a right angle in relation to its position at the time of forming. The reason for this was that the rough surface of the sample could reduce the strength measurement values. The test was carried out until the moment when the specimen was destroyed, i.e., when the transverse tensile strength of the concrete was exceeded.

**Figure 3:**
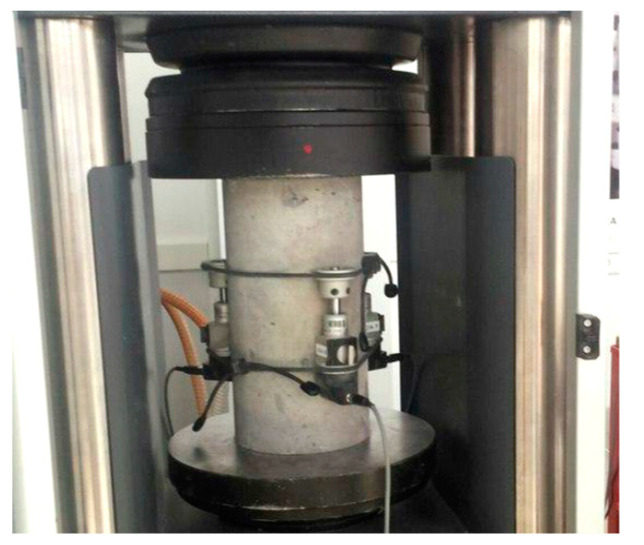
Test setup for testing the static modulus of elasticity of reference concrete (C-REF).

The cylindrical samples were tested for the static modulus of elasticity, according to the standard EN 12390-13 [55]. Figure 3 shows the test setup on which the reference concrete was tested. First, the height spots were determined on the cylindrical sample. Then, the strain gauges were placed in the marked places at equal intervals in order to measure the deformation. Afterwards, the sample was placed in a testing machine, equipped with a hydraulic press. The tests of the modulus of elasticity were carried out several times after the initial loading and unloading, in order to exclude the immediate permanent deformations. The specimen was then subjected to the load and unload, for several times. The modulus of elasticity of the cylindrical samples made of the fiber concrete was tested with sand caps, the cylinders were compressed through layers of sand.

**Figure 4:**
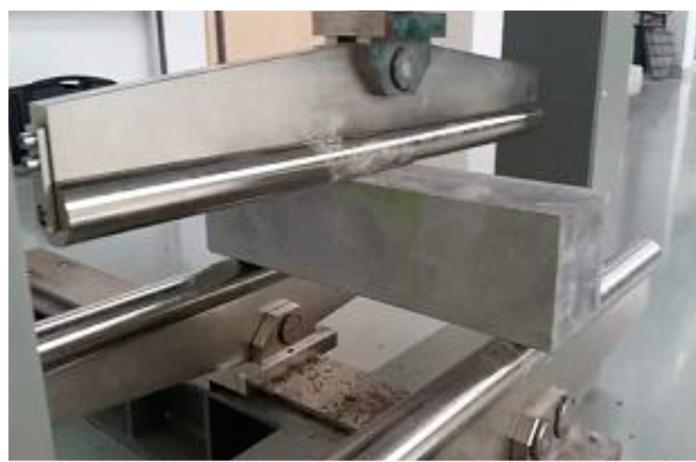
Flexural strength test of C-REF.

The bending tensile strength test was carried out on the basis of the EN 12390-5 standard [56]. The samples used for the tests had the shape of a prism of the dimensions given above. The test setup is shown in Figure 4.

After the samples were saturated with water, they were tested for frost resistance. The samples were frozen for 4 h at −20 °C and subsequently thawed in water at +20 °C. The samples were evaluated in terms of mass loss and reduction in compressive strength, in comparison to the reference concrete, which was submerged in water +20 ± 2 °C throughout the test. The test was carried out in line with the PN-B-06250 standard [57].

The beam specimens were placed in the testing machine and then subjected to a central force load. The essence of the research was to determine the stresses in the tensile zone that caused damage to the specimen. The flexural strength was considered to be the moment when an increase in the loading force did not increase the load-bearing capacity of the element.

**Figure 5:**
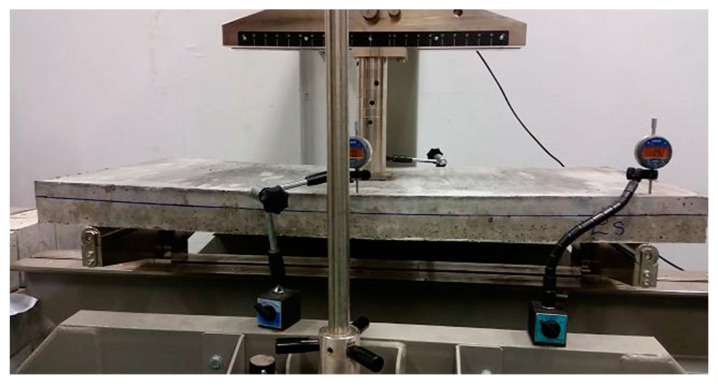
Bending resistance test of a concrete slab—specimen sample subjected to a local axial force.

The bending strength test of the plate elements was carried out on the slabs with reinforced fiber concrete compression zone and the slabs made entirely of the reference concrete. The reinforced concrete slabs were placed in the CONTROLS (Controls S.p.A., Milan, Italy) testing machine, where they were loaded locally with axial force through a centrally positioned steel plate (Figure 5).

**Figure 6:**
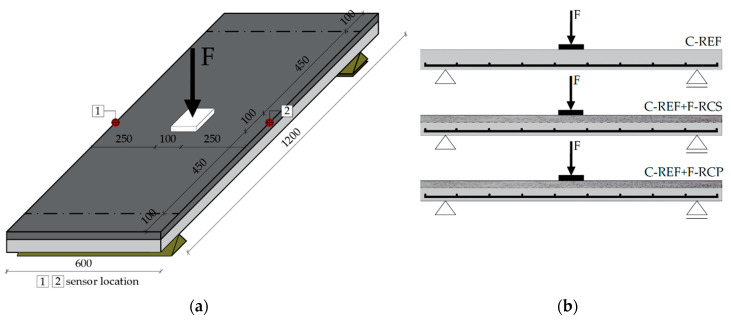
Bending resistance test—scheme of the tested slabs: (**a**) cavalier perspective; (**b**) cross-section.

Inductive sensors were also set up to measure the displacement during the tests. Sensors no. 1 and no. 2 were located in the center of the longer edges of the slab (Figure 6). Figure 6 shows a scheme of the tested slabs.

The induction sensors recorded the actual deflections of the composite and control slabs. The readout was performed at the initial phase, and then each time the applied force increased by 5 kN. The force that destroyed the tested element was assumed as the bearing capacity of the slab. Each tested slab element was destroyed.

**Figure 7:**
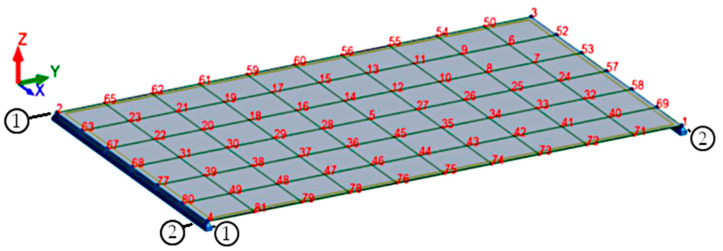
The numerical model of the slab element.

In order to compare the results of deflections obtained from experimental studies, a model of the research element was designed in the Autodesk Robot Structural Analysis numerical program, which is based on the finite element method. In the calculating program, a slab made of the reference concrete with the parameters obtained from the experimental research was modeled (the length of the slab between supports amounted to 1000 mm). Then, the modeled slab was divided into finite elements with a mesh size of 10 cm (Figure 7). Afterwards, the slab was loaded with its weight and a centric axial force of 10 kN, 15 kN, 20 kN and 25 kN.

## 3. Results and Discussion

**Figure 8:**
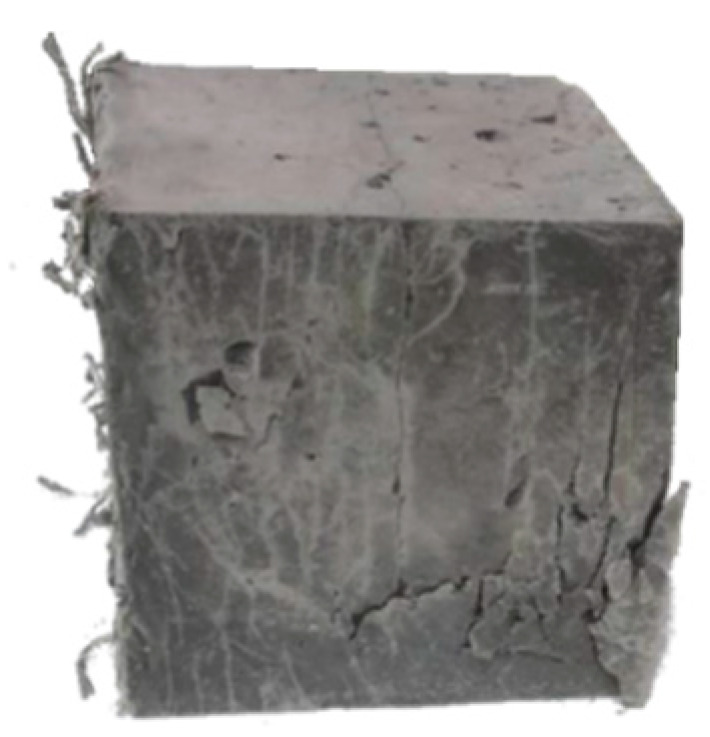
Destroyed fiber-reinforced concrete with polypropylene fibers (F-RCP) cubic specimen after the strength test.

**Figure 9:**
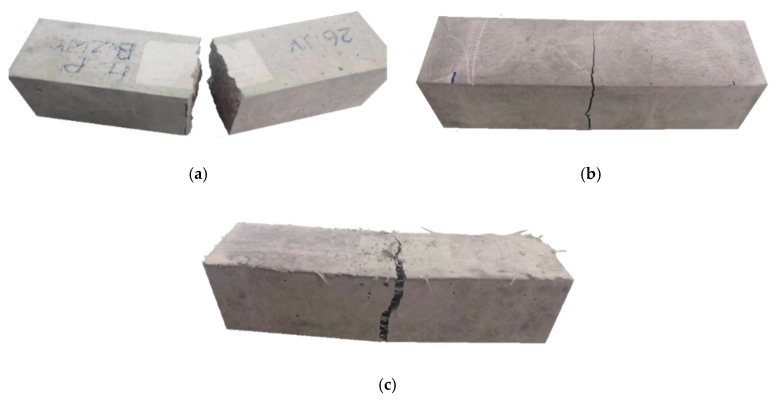
View of the destroyed sample after the flexural strength test: (**a**) reference concrete; (**b**) fiber-reinforced concrete with steel fibers and (**c**) fiber-reinforced concrete with polypropylene fibers.

The results of strength tests are summarized in Table 9. Figure 8 shows the appearance of the F-RCP sample after the compression strength test, while Figure 9 presents the concrete samples after the flexural strength test.

The studies on the fiber-reinforced samples showed that the use of fibers noticeably improved the mechanical properties of concrete compared to the concrete without them.

As it can be seen in Table 9, the highest compressive strength was achieved by the F-RCS concrete. The value of the compressive strength was 63.4% higher than C-REF. On the basis of the mean compressive strength values (Table 9), it could be concluded that the mean compressive strength of steel fiber concrete with 1% fiber content was 22.8% higher than that of polypropylene fiber concrete with the same fiber content. The low strength value in the case of the reference concrete may be caused by a high w/c ratio of 0.7. Developing the w/c ratio at an appropriate (low) level ensures that the properties of the concrete were favorably influenced, with particular emphasis on durability. In hardened concrete, as the w/c ratio decreased, the porosity (mainly capillary) decreased, which made the migration of aggressive liquid and gases into the concrete structure difficult. Increasing the w/c ratio in the concrete mix results in more pores in the concrete; thus the compressive strength was reduced. The tighter structure of the concrete matrix translates into higher concrete strength and also provides increased resistance to chemical aggression. Besides, a lower w/c ratio results in lesser shrinkage. Owing to a lower w/c ratio of 0.24, the F-RCS and F-RCP concretes achieved 1.6 and 1.3 times higher strength than C-REF, respectively. The ability of the fibers to delay the crack growth, inhibit crack propagation and reduce stress concentration at the tip of the crack is another related effect. Moreover, the flexural strength and modulus of elasticity were also higher for the concretes with added fibers −17.4% and 10.4% for F-RCS; 27.2% and 9.0% for F-RCP compared to C-REF, respectively (Table 9).

While analyzing the results only for the fiber-reinforced concretes, which had the same percentage of fibers added, it can be seen that the compressive strength of the F-RCS concrete was approximately 1.2 times higher than the F-RCP concrete. After the analysis of the results, it can be concluded that this value was influenced to a greater extent by steel fibers. The effectiveness of steel fibers lies in their dimensions. Shorter fibers, as is the case here, are more effective in preventing the growth and spread of microcracks. The longer fibers, with higher aspect ratio, are more effective for macrocracks and, as a result, in improving the compressive strength.

The volume and distribution of the fibers used could also reduce the compressive strength of the F-RCP samples. Although in both concretes the fibers were added in the same amount by weight, there were much more polypropylene fibers by volume. The polypropylene fibers per m^3^ amounted to 22.17 dm^3^ while steel fibers −2.94 dm^3^·m^−3^. Air voids can be formed by long fibers with a relatively high fiber content, which can also reduce the compressive strength. Figure 8 shows a specimen made of F-RCP after the compressive strength test.

**Figure 10:**
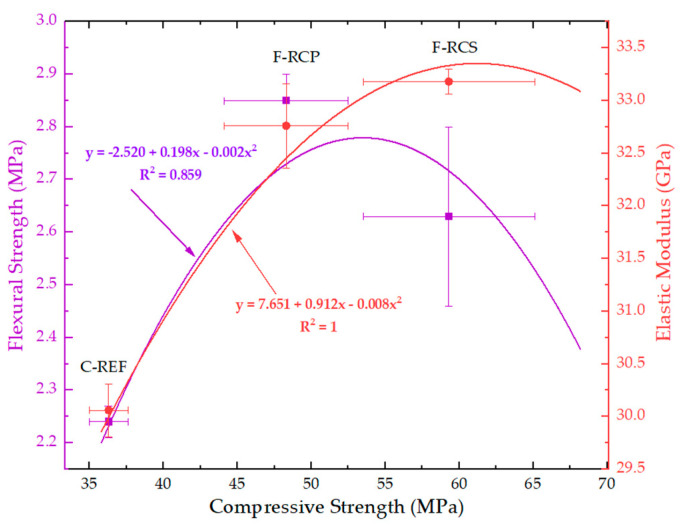
Correlation between compressive strength, the elastic modulus of concrete and flexural strength.

Concerning the flexural strength, a different situation occurred. In this case, the higher flexural strength was achieved by the specimens made of the polypropylene fiber-reinforced concrete. Despite the lower strength parameters of PP compared to SF, the F-RCP sample achieved 8.4% higher strength value, compared to F-RCS (Table 9, Figure 10). Under load, short SF only managed to eliminate microcracks, whereas due to their length and volume (22.17 dm^3^·m^−3^), the PP could carry macrocracks as can be seen in Figure 9c.

The value of the modulus of elasticity was strongly linked to the compressive strength. The F-RCS samples reached a higher value in the case of the elastic modulus. The value of the module for them was 1.01 times higher than in the case of F-RCP (Table 9, Figure 10). Aggregates, cement paste and ITZ are the three phases forming concrete and the modulus of elasticity depends primarily on the elasticity moduli of these phases and their volume [58]. Thus, for F-RCP, the modulus of elasticity decreases due to the strength properties of the fibers (Table 1), their length and their volume per m^3^.

The correlation between the compressive strength and flexural strength as well as the correlation between compressive strength and modulus of elasticity is shown in Figure 10.

**Figure 11:**
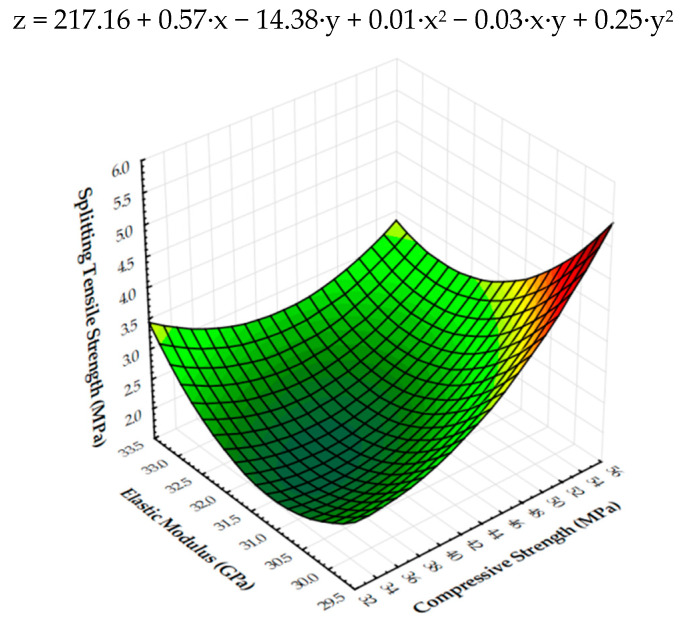
Three-dimensional surface plot of flexural strength (MPa) against compressive strength (MPa) and elastic modulus (GPa).

Additionally, the splitting tensile strength against the compressive strength and elastic modulus is shown in Figure 11. This correlation is described by the equation of the plane shown also in Figure 11. The flexural strength is the dependent variable in this graph. It is expressed by two variables, which are independent: x—compressive strength and y—modulus of elasticity. Figure 11 shows—taking into account the results obtained in the work—that with the increase in the compressive strength and the decrease in the modulus of elasticity, a simultaneous increase in the flexural strength occurred.

In their work, Afroughsabet and Ozbakkaloglu [59] analyzed high strength concrete containing a different percentage of SF (hook ended) and PP. The fibers used had the following length: 60 mm SF and 12 mm PP. They were applied in various volume fractions. They also combined steel and polypropylene fibers (1% by volume of concrete) to test this combination. The tests conducted by Afroughsabet and Ozbakkaloglu showed that each of the concretes in which the fibers were used achieved higher strength parameters. The best results for the compressive strength were achieved by the concrete with the addition of 1% SF—after 91 days, the strength of the concrete sample was 104.3 MPa. The steel fiber concrete also showed better results in terms of the flexural strength, reaching a value of up to 61% higher compared to plain concrete. Ali et al. [60] used the coconut fiber as the main test subject. They added different amounts of fiber by weight to the mass of the cement and different lengths of fiber. The concrete that contained a higher fiber content or length achieved low moduli of elasticity, both static and dynamic. On the basis of their research, they came to the conclusion that the addition of 5% of 5 cm long coconut fibers contributed to the improvement of such properties as compressive toughness, compressive strength, modulus of rupture and flexural strength, but at the same time decreased the static modulus of elasticity, splitting tensile strength and density. The flexural strength increased by as much as 910%. On the other hand, the concrete with a fiber content of 2% and 3% with a length of 7.5 cm achieved a lower compressive strength than plain concrete. In the paper [61], the author examined the concrete with the addition of both SF and PP in various percentages. They also added the admixture of metakaolin in different percentages. Prasad concluded that the best strength results were achieved by the concrete with 15% metakaolin and 0.5% of SF and PP. The tensile, flexural and compressive strengths increased by 15.14%, 14.92% and 4.99%, respectively. However, with a fiber content of 1% and a 15% admixture, all values have decreased. Other authors [62] observed that SF increased the compressive strength of high performance concrete by about 3% at 1% fiber volume content, while PP reduced the compression strength by about 57% at 1% SF volume content. The concrete with 1% SF had the highest splitting tensile strength, higher by 55% than for the standard concrete. However, in their research, the addition of PP in the amount of 1% caused a rise in the splitting tensile strength by 14% and decrease in the static modulus of elasticity by 10%, compared to the standard high performance concrete. The static modulus of elasticity of the concrete with 1% PP was the lowest and 25% lower than that of the concrete with 1% SF. The microstructure investigations showed that the bond strength in the interfacial transition zone (ITZ) between PP and cement paste was poor as evidenced by a lot of pores between the PP and paste, which can be associated with a decrease in strength and modulus of elasticity [62].

In our tests (Table 9), the fibers significantly reduced the frost resistance of concrete. SF resulted in a 62% mass loss after 180 freezing–thawing cycles in comparison to REF concrete, while PP resulted in a five-fold decrease in concrete mass. Afroughsabet and Ozbakkaloglu [59] presented the beneficial effect of the SF and PP fibers after the freezing and thawing test. In the study by Barnat-Hunek et al. [20], the basalt fibers successfully protected the concrete against the damage of frost in contrast to the SF, which resulted in about 17% greater frost corrosion. In the research of Smarzewski and Barnat-Hunek [12], SF significantly increased the high performance concrete damage after 180 freezing–thawing cycles. After the test, the concrete had cracks on the surface and corrosion of the SF occurred in the concrete with the 1% SF content. The concrete with 1% PP had an 11% higher mass loss and concrete with 1% SF had 22 times greater mass loss than the concrete without any fibers [12]. The fibers in our study did not cause such concrete damage, because they were different than those in the tests [12]. The employed SFs have a length of 13 mm and a diameter of 0.16 mm, while the authors in [12,20] studied SF with a length of 50 mm and diameter 1 mm. They caused much larger weight losses of concrete. The conclusion is that in the aspect of the fiber concrete durability against frost, shorter SFs should be used, which will provide better frost resistance.

**Figure 12:**
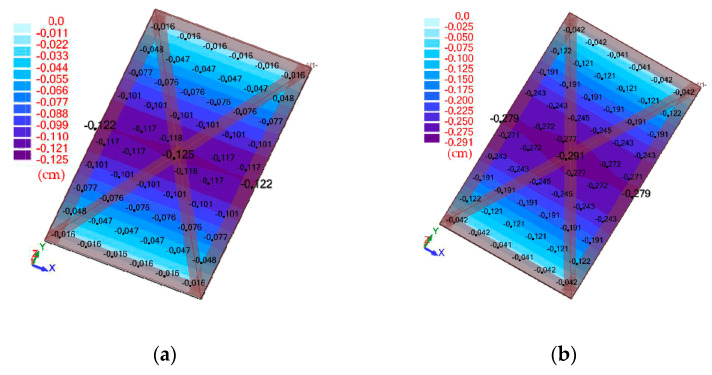

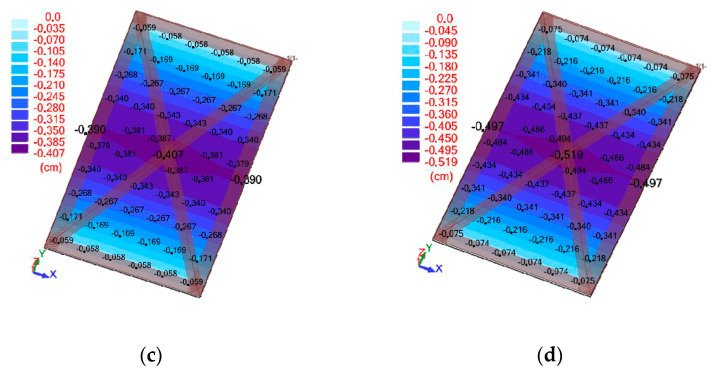
Map of deflections corresponding to the loading force: (**a**) 10 kN; (**b**) 15 kN; (**c**) 20 kN and (**d**) 25 kN.

Figure 7 mentioned above shows a numerical model of a slab made of reference concrete with the following parameters obtained from the experimental tests: exposure class XC1; slab thickness 8 cm; reinforcing steel grade B500SP; f_yk_ = 500 MPa; diameter of reinforcing bars 8 mm; f_ck_ = 36.3 MPa; modulus of elasticity of concrete 30.06 GPa; Poisson’s coefficient 0.2 and specific gravity 24.53 kN·m^−3^. Next, the slab was loaded with its weight and a centric axial force of 10 kN, 15 kN, 20 kN and 25 kN, and deflections were verified (Figure 12).

**Figure 13:**
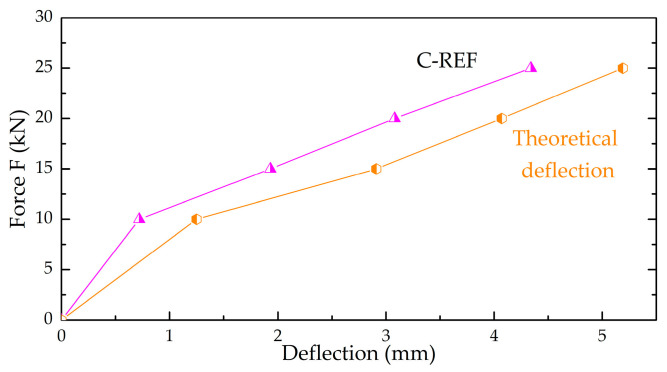
A graph showing a comparative analysis of experimental results (C-REF) and numerical deflection.

Figure 13 shows a comparison of the results obtained from the experimental studies and the numerical method.

Based on the values obtained, it could be concluded that with the increase in strength, the deflections obtained from the numerical program were greater than the experimental deflections for the slab made of the reference concrete. It was noted that the difference in results gradually decreased as the load increased. C-REF achieved a deflection of 20% less at 25 kN than its numerical model, and with a 5 kN load, the deflections were 74% lower. This shows that with the same parameters, the computer program will give higher deflection values. Relying solely on the computer calculations can lead to an erroneous analysis of the tested material and therefore experimental research is necessary.

**Figure 14:**
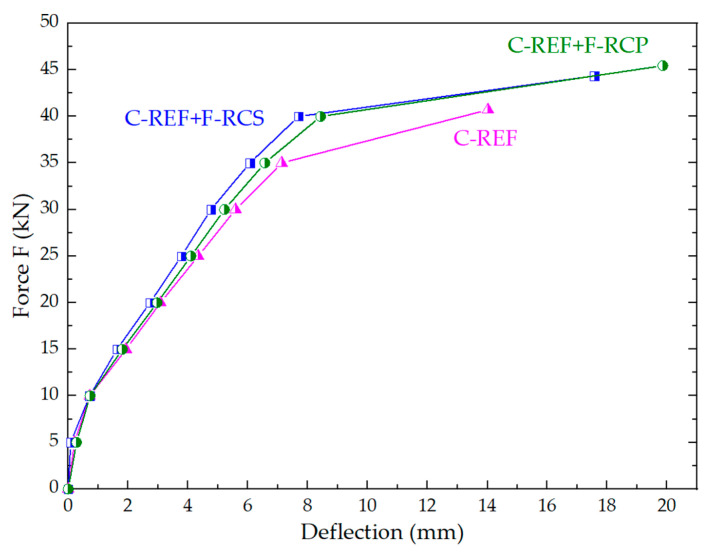
Comparison of the deflections of the tested slabs.

Table 10 and Figure 14 show the average values of deflections and average ultimate load of the tested slab elements.

The fastest loss of the bearing capacity was found in the reinforced concrete slab, which does not have a strengthened compression zone. Its load capacity corresponded to an ultimate load of 41.01 kN. It was 12% lower than the C-REF + F-RCP slab for which the ultimate load was the highest of all and amounted to 45.14 kN. The first crack on the C-REF slab was already observed with a load of about 20 kN. Figure 14 shows that the deflection range of a fully reinforced concrete element was much smaller than that of the composite models, even though the individual deflection values in relation to the corresponding forces were close to each other. The greatest deflections was observed in the beams made of the concrete with PP fiber addition, slightly lesser in the concrete with steel fiber, whereas the smallest deflections were observed in the case of the control beams made of non-modified concrete.

This means that more force is needed to destroy a slab with a strengthened compression zone. The reason for the possibility of obtaining a higher deflection value is the fact that the crack reaching the fiber concrete layer is temporarily stopped by the applied fibers. The stresses around the crack are transferred through the fiber from one side of the crack to the other, reducing the opening of the crack, thus increasing the load-bearing capacity of the component. After crossing the lower surface of the concrete with fibers, it was noticed that the application of loads was slower concerning the C-REF slab. The justification is the process of gradual pulling of fibers from the concrete matrix. The comparative analysis of the deflections and load capacities of composite slabs in relation to the slabs made of the reference concrete is presented in Table 10. On the basis of the data presented in Table 10, it was noted that the composite slabs with fiber-reinforced concrete with polypropylene fiber (1% by weight of cement) in the upper layer achieved a load capacity 12% higher with respect to the reference slabs. In the case of C-REF + F-RCS slabs, the strength increased by 9%, compared to the C-REF slabs. The highest bearing capacity was reached by the C-REF + F-RCP slab—45.14 kN. When comparing the C-REF + F-RCS and C-REF + F-RCP slabs, it can be seen that the deflections achieved by them during destruction differed by 13%, but the ultimate load differed only by 3%. The use of the fiber-reinforced concretes in the compression zone of the bent slab elements improved the strength properties of the slabs.

**Figure 15:**
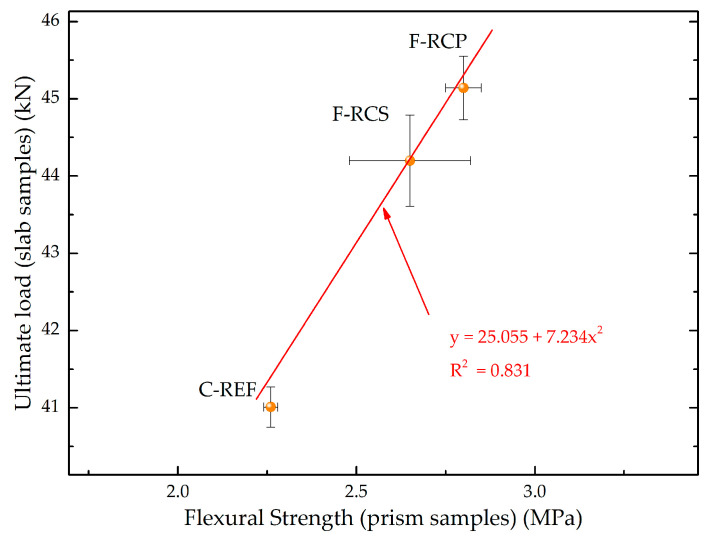
Correlation between flexural strength and ultimate load.

Figure 15 shows the correlation between the flexural strength of the prism samples (C-REF, F-RCS and F-RCP) and ultimate load under which the slab samples were destroyed. As can be seen in Figure 15, the highest force was transferred by those slabs in which fibers were used, the addition of which contributed to the highest flexural strength of the prism samples.

**Figure 16:**
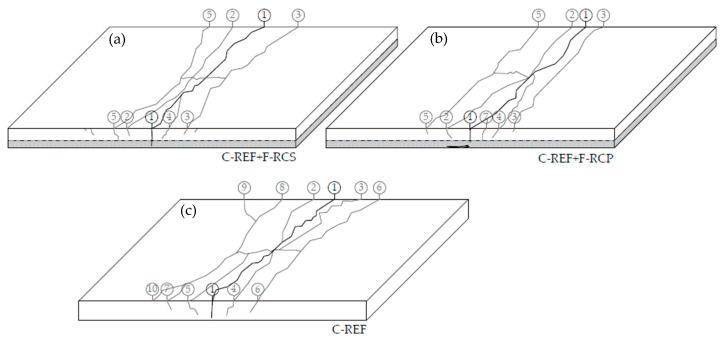
Scheme of crack development in the final testing phase of the slabs (**a**) C-REF + F-RCS slab; (**b**) C-REF + F-RCP slab; (**c**) C-REF slab.

The diagrams of the crack development in the tested slabs were illustrated based on the photographic documentation made during the research (Figure 16). The propagation of cracks was photographed following each time the applied force increased by 5 kN. The changes were marked on the slab samples, marking the value of force corresponding to the crack occurrence.

**Figure 17:**
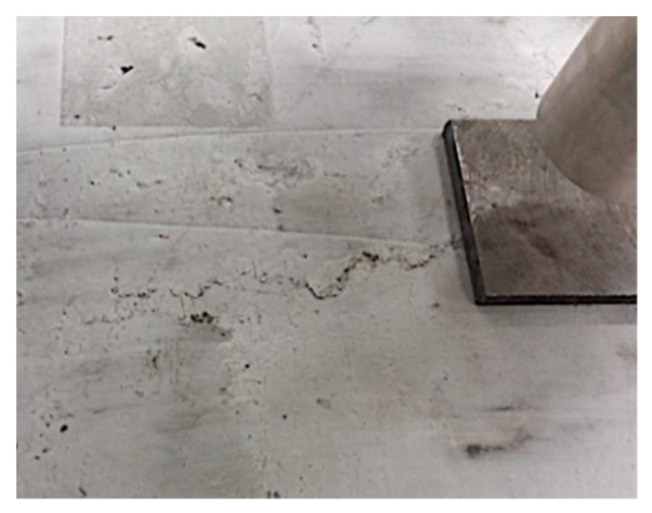
Compression zone of the damaged C-REF + fiber-reinforced concrete with steel fibers (F-RCS) slab element.

The maximum load capacity of the slabs of the C-REF + F-RCS series corresponded to the ultimate load of 44.34 kN. In the tested slabs, the first crack appeared at a load of about 26 kN. It was observed in the middle of the span of the slab. Under the influence of increasing load, further cracks appeared, parallel to the direction of the first crack (Figure 16). The development of cracks was along the whole width of the slab. It was observed that only the first crack tended to increase its width as the applied loads increased. The largest width of the crack in the tension zone of the element was 7 mm. In the compressed zone, after the element was destroyed, local crushing was observed on the surface of the slab (Figure 17).

The maximum load capacity of the slabs of the C-REF + F-RCP series corresponded to an ultimate load of 45.44 kN. It was observed that during the cyclic loading, the deflections of the tested slab element were slightly smaller in relation to the deflections of the slab using the fiber concrete with SF. However, the slab deflection limits were very close to each other. In the slab strengthened in the compression zone with the PP concrete, the first crack appeared under a 29 kN load. Moreover, as in the case of the composite slabs with the fiber concrete with the addition of SF, the largest crack was crack no. 1. Additional cracks (Figure 16) appeared with increasing loads, which were directed parallel to crack no. 1. The largest width of crack no. 1 was 11 mm. The high-modulus steel fiber reinforced the slabs when small and medium cracks occur. The low modulus PP, on the other hand, developed its full reinforcement capacity for large cracks.

In the case of reinforced concrete slabs of the C-REF series, the value of the ultimate load was 40.71 kN. On this basis, it could be concluded that the slabs achieved a lower bending capacity than the composite slabs. The values of recorded deflections of the reinforced concrete slabs were also lower and amounted to about 14 mm. This is an additional indication of a faster loss of the bearing capacity of the bending element. From the observations made during the tests and from the photographic documentation it was concluded that the slab made entirely of the reference concrete scratched at a load of 20 kN. The scheme of development of the cracks is shown in Figure 16. The number of cracks was twice as high as for the strengthened slabs in the compression zone. The development and the width of cracks were more expressive in relation to the composite slabs. Cracks no. 1, 2, 3, 4 and 5 increased in width along with the applied load. The largest width was reached by crack no. 1, which was 16 mm. Additionally, many small cracks, deviating from the “main” ones, were created.

Wang et al. [63] analyzed the composite slabs made of the reinforced concrete and SF. They made four different types of slabs with reinforcement in different configurations. In three of them, the core layer was concrete (200 kg·m^−3^), in the fourth type, the core and the compressive layer was concrete with a density of 700 kg·m^−3^. The reinforcement was in different fiber and bar configurations. On the basis of their tests, it turned out that the highest force was carried by the slab B2—59 kN, achieving a deflection under its influence of 38 mm. The first cracks appeared under 20 kN and the final deflection was 51 mm. The B2 slab was composed of 48 kg of steel (0.46% on the tensile side) and the compressive and tensile layers were reinforced with steel rods. Mansour et al. [64] divided their research into two stages. In the first one, they selected the optimum percentage of steel fibers of 1%, based on research. In the second stage, they made four concrete slabs—three of them were surface reinforced with a layer of concrete with steel fibers. It was laid after the previous layer of concrete with traditional reinforcement had hardened. Before the fiber-reinforced concrete layer was laid, the concrete surface was roughened in three different ways. A reinforcement layer in the form of the traditionally reinforced concrete was laid on the reference slab. The research showed that the concrete the reinforcement layer of which was made of the concrete with traditional reinforcement reached the highest ultimate load-carrying capacity. It was 7.5% higher than the other three slabs. However, the addition of steel fibers contributed to 17.5% less deflection at the ultimate load than the reference slab. Abdullah [65] made 14 slabs in his research. He divided the slab samples into six groups. One group included a reference sample and the next five groups of samples differed in the thickness of the strengthening layer, percentage of SF, compression strength of ferrocement and the number of strengthening layers (in different combinations). He concluded that the main factor influencing the strength of the strengthening layer is SF. The highest load capacity, i.e., 57.62 kN, was achieved by a slab with three layers of the reinforcing layer, for which a single layer of ferrocement had a compressive strength of 40 MPa and 0.75% of the SF content. At this force, the deflection was only 3.2 mm. The main object of the research by Frazão et al. [66] was sandwich panels consisting of lightweight fiber-reinforced concrete and sisal fiber-cement composites. The core of the panels was lightweight concrete with PP (60 mm) and the exterior layers were sisal fiber-cement composite laminates (2 × 15 mm)—four contained short sisal fibers (50 mm) and four other long sisal fibers (700 mm). The research in the paper showed that the use of long sisal fibers is more effective. They improved the tensile and flexural strength of the sandwich panels. They showed more cracks, but the gap between cracks was much smaller.

The study was conducted using the fibers produced by a service company; however, they differ from typical short fibers most often used in the concrete technology, which have the length of 12 mm, diameter of 25 µm and density of 0.9 g/cm^3^. Additionally, they show the tendency towards clustering, which hinders their distribution in the concrete mix [12,62]. The fibers that resemble the recycled PP fibers in terms of appearance and dimensions were employed as well. They can be obtained, e.g., from shredded PET bottles or other PVC. The directions for further sustainable research could involve the influence of dimensions, length, diameter and type of recycled fiber on the strength properties and durability of fiber-reinforced concrete as well as slabs made of this material.

## 4. Conclusions

This work aimed to demonstrate the effect of combining a fiber concrete layer with ordinary concrete on improving the strength characteristics of the composite slab structural elements in relation to the slab elements made entirely of the non-fiber concrete. The research program and the analysis of the obtained results allow drawing the following conclusions:

- The compressive strength tests of the cubic samples made of fiber-reinforced concrete with SF and PP fibers of the same content showed that the average compressive strength of F-RCS was higher than that of F-RCP by 23%. The reduction in the average compressive strength of F-RCP concrete was caused by excessive fiber volume. Higher compressive strength of the reference concrete could also be achieved by changing the water–cement ratio.
- The flexural strength tests showed that the average strength of the samples made of the C-REF concrete reached a lower value in relation to the average strength of the samples made of the F-RCS and F-RCP fiber-reinforced concrete by 15% and 27%, respectively. Under the maximum loading force, the destruction of the beam made of the C-REF concrete took on the character of a sudden brittle crack. In the case of the beams made of the fiber-reinforced concrete with the addition of steel and polypropylene fibers, a crack was formed at a certain value of force, which did not cause a loss of strength. Under the increasing force, the beam bent, showing a further load-bearing capacity.
- The bending resistance tests of the testing elements were carried out on the reinforced concrete slabs with a strengthened compression zone made of the fiber-reinforced and reinforced concrete slab made of normal concrete. In the scope of these tests, it was found that composite slabs achieved a higher load-bearing capacity, with respect to the slabs without a strengthened compression zone. It was noted that the composite slabs with fiber-reinforced concrete with polypropylene fibers in the upper layer achieved a load capacity 12% higher with respect to the reference slabs. In the case of the fiber-reinforced concrete slabs with steel fibers, the strength increased by 9% compared to the C-REF slabs.
- In the composite slabs, the development of cracks was all over their width, while only the first crack tended to increase in width along with the loads applied. In the case of the reference plates, the development and width of cracks were more expressive. Several cracks increased in width along with the load. It was observed that more force was needed to destroy a slab with a strengthened compression zone.
- The analysis of the results obtained from the experimental studies showed that the application of the fiber-reinforced concrete layer in the compression zone of the bending slabs allowed increasing their load capacity and stiffness. This confirmed the effectiveness of using of fiber-reinforced concrete to improve the strength characteristics of bending slab elements.

## Figures and Tables

**Table 1:** Properties of the fibers applied.

Type of Fibers	Fiber Length	Fiber Diameter	Tensile Strength	Modulus of Elasticity
	(mm)	(mm)	(MPa)	(GPa)
steel	13	0.16	300	200
polypropylene	50	0.72	600	5

**Table 2:** Composition of concrete mixtures.

Components	Unit	C-REF	F-RCS	F-RCP
CEM I 42.5R cement	(kg∙m^−3^)	260	–	–
CEM I 52.5 HSR cement	(kg∙m^−3^)	–	720	720
Quartz sand (0.2–0.8 mm)	(kg∙m^−3^)	–	900	900
Sand (0–2 mm)	(kg∙m^−3^)	730	–	–
Gravel (2–8 mm)	(kg∙m^−3^)	1134	–	–
Reactive powder	(kg∙m^−3^)	–	25.2	25.2
Silica ash	(kg∙m^−3^)	–	216	216
Superplasticizer	(kg∙m^−3^)	–	5.76	5.76
Plasticizing admixture	(kg∙m^−3^)	1.82	–	–
Steel fibers	(kg∙m^−3^)	–	20.4	–
Polypropylene fibers	(kg∙m^−3^)	–	–	20.4
Water	(kg∙m^−3^)	185	173	173

**Table 3:** Technical parameters of CEM I 42.5 R Portland cement (on the basis of the manufacturer’s information [45]).

Parameters	Unit	Value	Requirements of the Standard EN 197-1 [44]
Initial setting time	(min)	184	≥60
End of setting	(min)	242	–
Specific density	(g∙cm^−3^)	3.08	–
Specific surface	(cm^2^∙g^−1^)	4124	–
Loss on ignition	(%)	3.33	≤5
Compressive strength			
after 2 days	(MPa)	30.1	≥20
after 28 days		60.2	≥42.5 ≤62.5
Volume change	(mm)	1.0	≤10
SO_3_ content	(%)	2.95	≤4.0
Cl content	(%)	0.089	≤0.1
Insoluble residue	(%)	0.57	≤5

**Table 4:** Technical parameters of CEM I 52.5 HSR Portland cement (on the basis of the manufacturer’s information [46]).

Parameters	Unit	Value	Requirements of the Standard EN 197-1 [44]
Initial setting time	(min)	249	≥45
End of setting	(min)	320	–
Specific density	(g∙cm^−3^)	3.23	–
Specific surface	(cm^2^∙g^−^^1^)	4216	–
Water demand	(%)	29.80	–
Compressive strength			
after 2 days	(MPa)	30.6	≥20
after 28 days		60.9	≥52.5
SO_3_ content	(%)	2.66	≤4.0
Cl content	(%)	0.06	≤0.1

**Table 5:** Properties of polycarboxylate superplasticizer.

Density	pH	Contractual Dry Substance Content	Chloride Content	Alkaline Content
(kg∙dm^−3^)	(–)	(%)	(%)	(%)
1.08	4.0	40.0	≤0.1	≤0.5

**Table 6:** Properties of plasticizing admixture.

Raw Material Base	Density (+20 °C)	Form	Color	pH	Cl^−^	Na_2_O
(–)	(g∙cm^−3^)	(–)	(–)	(–)	(%)	(%)
lignosulfonates	4.0	liquid	dark brown	4.5	≤0.1	≤1.5

**Table 7:** Technical data of the reactive powder (on the basis of the manufacturer’s information [49]).

Parameters	Unit	Value
Form	–	loose powder
Composition		calcined kaolin (metakaolin)
Color	–	white–beige, cream
Specific gravity	(g∙cm^−3^)	2.6–÷ 2.63
Bulk density	(g∙cm^−3^)	0.6 ÷ 0.7
Fineness		1.4
Non-volatile component content	(%)	approx. 100
Solubility and miscibility with water	–	mixes in all proportions
Water demand	(mL)	300
pH value (aqueous solution/20 °C)	–	8–9
Melting temperature	(°C)	>900

**Table 8:** Silica ash properties [50].

SiO (Max)	H_2_O (Max)	Roasting Losses (Max)	C (Max)	Fe_2_O_3_ (Max)	Al_2_O_3_ (Max)	CaO (Max)	Specific Surface
(%)	(%)	(%)	(%)	(%)	(%)	(%)	(m^2^∙g^−1^)
85	1.5	4.0	4.0	4.0	1.5	1.0	15.0–35.0

**Table 9:** Mechanical properties of the concretes.

Type of Concrete/Descriptive Statistics		Compressive Strength	Flexural Strength	Elastic Modulus	Loss Mass after 180 Cycles F-T
		(MPa)	(MPa)	(GPa)	(%)
C-REF	Mean	36.5	2.26	30.12	0.23
	SD	1.3	0.03	0.25	0.02
	CV (%)	4	1	0.1	0.2
F-RCS	Mean	59.8	2.65	33.23	0.61
	SD	5.8	0.17	0.12	0.91
	CV (%)	10	6	0.4	0.3
F-RCP	Mean	48.7	2.80	32.65	1.24
	SD	4.2	0.05	0.4	0.3
	CV (%)	9	2	1.1	0.2

SD—standard deviation, CV—coefficient of variation.

**Table 10:** Average deflection for the concrete slabs tested.

Type of Concrete	Force F (kN)								Average Ultimate Load (kN)		
	5	10	15	20	25	30	35	40	41.01	44.20	45.14
	Deflection (mm)										
C-REF	0.21	0.72	1.93	3.08	4.34	5.59	7.13	–	14.02	–	–
C-REF + F-RCS	0.28	0.73	1.81	2.97	4.10	5.22	6.57	8.43	–	17.57	–
C-REF + F-RCP	0.20	0.46	1.60	2.58	3.59	4.59	5.61	7.11	–	–	19.86

## References

[1] Mousavi S.M., Ranjbar M.M., Madandoust R. (2019). Combined effects of steel fibers and water to cementitious materials ratio on the fracture behavior and brittleness of high strength concrete. Eng. Fract. Mech..

[2] Rashid M.U. (2020). Experimental investigation on durability characteristics of steel and polypropylene fiber reinforced concrete exposed to natural weathering action. Constr. Build. Mater..

[3] Chan R., Liu X., Galobardes I. (2020). Parametric study of functionally graded concretes incorporating steel fibres and recycled aggregates. Constr. Build. Mater..

[4] Thomas J., Ramaswamy A. (2007). Mechanical Properties of Steel Fiber-Reinforced Concrete. J. Mater. Civ. Eng..

[5] Pajak M., Ponikiewski T. (2013). Flexural behavior of self-compacting concrete reinforced with different types of steel fibers. Constr. Build. Mater..

[6] Groli G., Caldentey A.P. (2017). Improving cracking behaviour with recycled steel fibres targeting specific applications—Analysis according to fib Model Code 2010. Struct. Concr..

[7] Kim J.-J., Yoo D.-Y. (2019). Effects of fiber shape and distance on the pullout behavior of steel fibers embedded in ultra-high-performance concrete. Cem. Concr. Compos..

[8] De La Fuente A., Escariz R.C., De Figueiredo A.D., Molins C., Aguado A. (2012). A new design method for steel fibre reinforced concrete pipes. Constr. Build. Mater..

[9] Shen D., Liu X., Zeng X., Zhao X., Jiang G. (2020). Effect of polypropylene plastic fibers length on cracking resistance of high performance concrete at early age. Constr. Build. Mater..

[10] Di Maida P., Radi E., Sciancalepore C., Bondioli F. (2015). Pullout behavior of polypropylene macro-synthetic fibers treated with nano-silica. Constr. Build. Mater..

[11] Khan M., Ali M. (2018). Effectiveness of hair and wave polypropylene fibers for concrete roads. Constr. Build. Mater..

[12] Smarzewski P., Barnat-Hunek D. (2017). Effect of Fiber Hybridization on Durability Related Properties of Ultra-High Performance Concrete. Int. J. Concr. Struct. Mater..

[13] Khan M., Cao M., Ali M. (2020). Cracking behaviour and constitutive modelling of hybrid fibre reinforced concrete. J. Build. Eng..

[14] Pan J., Cai J., Ma H., Leung C.K.Y. (2018). Development of Multiscale Fiber-Reinforced Engineered Cementitious Composites with PVA Fiber and CaCO3 Whisker. J. Mater. Civ. Eng..

[15] Almusallam T., Ibrahim S., Al-Salloum Y., Abadel A., Abbas H. (2016). Analytical and experimental investigations on the fracture behavior of hybrid fiber reinforced concrete. Cem. Concr. Compos..

[16] James A.F., Rasool M.A., Genesh S. (2017). Effect of hair fibre and GGBS on various properties of concrete–an experimental study. Int. J. Civ. Eng. Technol..

[17] A Zaidi S.K. (2018). An Experimental Study on Human Hair Fiber Reinforced Concrete. Trends Civ. Eng. Arch..

[18] Barnat-Hunek D., Szymańska-Chargot M., Jarosz-Hadam M., Łagód G. (2019). Effect of cellulose nanofibrils and nanocrystals on physical properties of concrete. Constr. Build. Mater..

[19] Brzyski P., Barnat-Hunek D., Suchorab Z., Łagód G. (2017). Composite Materials Based on Hemp and Flax for Low-Energy Buildings. Materials.

[20] Barnat-Hunek D., Góra J., Andrzejuk W., Łagód G. (2018). The Microstructure-Mechanical Properties of Hybrid Fibres-Reinforced Self-Compacting Lightweight Concrete with Perlite Aggregate. Materials.

[21] Li M., Gong F., Wu Z. (2020). Study on mechanical properties of alkali-resistant basalt fiber reinforced concrete. Constr. Build. Mater..

[22] High C., Seliem H.M., El-Safty A., Rizkalla S.H. (2015). Use of basalt fibers for concrete structures. Constr. Build. Mater..

[23] Kimm M., Pico D., Gries T. (2019). Investigation of surface modification and volume content of glass and carbon fibres from fibre reinforced polymer waste for reinforcing concrete. J. Hazard. Mater..

[24] Pereira E.L., Junior A.L.D.O., Fineza A.G. (2017). Optimization of mechanical properties in concrete reinforced with fibers from solid urban wastes (PET bottles) for the production of ecological concrete. Constr. Build. Mater..

[25] Khan S.U., Ayub T. (2020). Flexure and shear behaviour of self-compacting reinforced concrete beams with polyethylene terephthalate fibres and strips. Structures.

[26] Shaikh F.U.A., Luhar S., Arel H.Ş., Luhar I. (2020). Performance evaluation of Ultrahigh performance fibre reinforced concrete—A review. Constr. Build. Mater..

[27] Merli R., Preziosi M., Acampora A., Lucchetti M.C., Petrucci E. (2020). Recycled fibers in reinforced concrete: A systematic literature review. J. Clean. Prod..

[28] Alzubaidi R., Barakat S., Al Toubat S. (2013). Effects of adding brass byproduct on the basic properties of concrete. Constr. Build. Mater..

[29] Ma W., Qin Y., Li Y., Chai J., Zhang X., Ma Y., Liu H., Junrui C. (2020). Mechanical properties and engineering application of cellulose fiber-reinforced concrete. Mater. Today Commun..

[30] Li Z., Wang X., Wang L. (2006). Properties of hemp fibre reinforced concrete composites. Compos. Part A: Appl. Sci. Manuf..

[31] Banthia N., Gupta R. (2004). Hybrid fiber reinforced concrete (HyFRC): Fiber synergy in high strength matrices. Mater. Struct..

[32] Afroughsabet V., Biolzi L., Ozbakkaloglu T. (2016). High-performance fiber-reinforced concrete: A review. J. Mater. Sci..

[33] Choi J.-I., Song K.-I., Song J.-K., Lee B.Y. (2016). Composite properties of high-strength polyethylene fiber-reinforced cement and cementless composites. Compos. Struct..

[34] Zhang P., Li Q.-F. (2013). Effect of polypropylene fiber on durability of concrete composite containing fly ash and silica fume. Compos. Part B: Eng..

[35] Colombo I.G., Colombo M., Di Prisco M. (2015). Tensile behavior of textile reinforced concrete subjected to freezing–thawing cycles in un-cracked and cracked regimes. Cem. Concr. Res..

[36] Bagherzadeh R., Sadeghi A.-H., Latifi M. (2011). Utilizing polypropylene fibers to improve physical and mechanical properties of concrete. Text. Res. J..

[37] Grzymski F., Musiał M. (2017). Testing methodology of fiber–reinforced concrete mechanical properties. Builder.

[38] Kalpana M., Tayu A. (2020). Experimental investigation on lightweight concrete added with industrial waste (steel waste). Mater. Today: Proc..

[39] Sadowska–Buraczewska B. (2011). New generation concretes as a strengthening layer in beam bending elements. Civ. Environ. Eng..

[40] Lapko A., Sadowska-Buraczewska B., Tomaszewicz A. (2005). Experimental and numerical analysis of flexural composite beams with partial use of high strength/high performance concrete. J. Civ. Eng. Manag..

[41] Peng Y., Wu C., Li J., Liu J., Liang X. (2019). Mesoscale analysis on ultra-high performance steel fibre reinforced concrete slabs under contact explosions. Compos. Struct..

[42] Niwa J., Fakhruddin, Matsumoto K., Sato Y., Yamada M., Yamauchi T. (2016). Experimental study on shear behavior of the interface between old and new deck slabs. Eng. Struct..

[43] European Committee for Standardization (2016). EN 206+A1:2016–12. Concrete—Part 1: Specification, Performance, Production and Conformity.

[44] European Committee for Standardization (2012). EN 197–1:2012. Cement—Part 1: Composition, Specifications and Conformity Criteria for Common Cements.

[45] Cement CZERWONY | CEMEX Polska. https://www.cemex.pl/cement–czerwony.aspx.

[46] Cement HSR 52,.5—Oferta producenta—Lafarge. https://www.lafarge.pl/cement–hsr–525–archiwum.

[47] European Committee for Standardization (2010). EN 12620+A1:2010. Aggregates for Concrete.

[48] European Committee for Standardization (2004). EN 1008:2004. Mixing Water for Concrete—Specification for Sampling, Testing and Assessing the Suitability of Water, Including Water Recovered from Processes in the Concrete Industry, as Mixing Water for Concrete.

[49] Astra MK 40—Astra. https://www.astra–polska.com/oferta/betony–przemyslowe/astra–mk–40/.

[50] Huta Łaziska SA SILIMIC®. http://hlsili.pl/oferta/silimic/.

[51] European Committee for Standardization (2013). EN 12390–1:2013–03. Testing Hardened Concrete—Part 1: Shape, Dimensions and Other Requirements for Specimens and Moulds.

[52] European Committee for Standardization (2019). PN–EN 12390–2:2019–07. Testing Hardened Concrete—Part 2: Making and Curing Specimens for Strength Tests.

[53] European Committee for Standardization (2008). EN 1992–1–1:2008. Eurocode 2: Design of Concrete Structures—Part 1–1: General Rules and Rules for Buildings.

[54] European Committee for Standardization (2019). EN 12390–3:2019–07. Testing Hardened Concrete—Part 3: Compressive Strength of Test Specimens.

[55] European Committee for Standardization (2014). EN 12390–13:2014–02. Testing Hardened Concrete—Part 13: Determination of Secant Modulus of Elasticity in Compression.

[56] European Committee for Standardization (2019). EN 12390–5:2019–08. Testing Hardened Concrete—Part 5: Flexural Strength of Test Specimens.

[57] Polish Committee for Standardization (1988). PN–B–06250:1988. Ordinary Concrete (In Polish).

[58] Nadeau J. (2003). A multiscale model for effective moduli of concrete incorporating ITZ water–cement ratio gradients, aggregate size distributions, and entrapped voids. Cem. Concr. Res..

[59] Afroughsabet V., Ozbakkaloglu T. (2015). Mechanical and durability properties of high-strength concrete containing steel and polypropylene fibers. Constr. Build. Mater..

[60] Ali M., Liu A., Sou H., Chouw N. (2012). Mechanical and dynamic properties of coconut fibre reinforced concrete. Constr. Build. Mater..

[61] Prasad D.H. (2020). An Experimental Study on Compressive Stength of Composite Fiber Reinforced Concrete with Metakaolin as Admixture. Int. J. Res. Appl. Sci. Eng. Technol..

[62] Smarzewski P., Barnat-Hunek D. (2017). Property Assessment of Hybrid Fiber-Reinforced Ultra-High-Performance Concrete. Int. J. Civ. Eng..

[63] Wang Y., Liu H., Xi C., Dou G., Qian L. (2019). Static Analysis of Properties of a Composite Slab Made from Steel Fibers and a Reinforced Foam Concrete. Mech. Compos. Mater..

[64] Mansour F.R., Abu Bakar S., Vafaei M., Alih S.C. (2017). Effect of Substrate Surface Roughness on the Flexural Performance of Concrete Slabs Strengthened with a Steel-Fiber-Reinforced Concrete Layer. PCI J..

[65] Abdullah M.D. (2018). Experimental and Theoretical Behavior of Reinforced Concrete Two Way Slabs Strengthened by Steel Fiber Ferrocement Layers at Tension Zone. J. Univ. Babylon Pure Appl. Sci..

[66] Frazão C., Barros J., Filho R.D.T., Ferreira S., Gonçalves D. (2018). Development of sandwich panels combining Sisal Fiber-Cement Composites and Fiber-Reinforced Lightweight Concrete. Cem. Concr. Compos..

